# Epidemiology of Anorexia Nervosa in Men: A Nationwide Study of Finnish Twins

**DOI:** 10.1371/journal.pone.0004402

**Published:** 2009-02-10

**Authors:** Anu Raevuori, Hans W. Hoek, Ezra Susser, Jaakko Kaprio, Aila Rissanen, Anna Keski-Rahkonen

**Affiliations:** 1 Department of Public Health, University of Helsinki, Helsinki, Finland; 2 New York State Psychiatric Institute, New York, New York, United States of America; 3 Parnassia Bavo Psychiatric Institute, The Hague, The Netherlands; 4 Department of Psychiatry, University Medical Center Groningen, University of Groningen, Groningen, The Netherlands; 5 Department of Epidemiology, Mailman School of Public Health, Columbia University, New York, New York, United States of America; 6 Department of Mental Health, National Public Health Institute, Helsinki, Finland; 7 Obesity Research Unit, Department of Psychiatry, Helsinki University Central Hospital, Helsinki, Finland; Universidad Nacional Mayor de San Marcos, Peru

## Abstract

**Background:**

To examine the epidemiology of anorexia nervosa in men, we screened Finnish male twins born in 1975–79.

**Methods and Findings:**

Men (N = 2122) from FinnTwin16 birth cohorts were screened for lifetime eating disorders by a questionnaire. The screen positives (N = 18), their male co-twins (N = 10) and those with lifetime minimum BMI≤17.5 (N = 21) were administered the Structured Clinical Interview for DSM-IV anorexia nervosa. The incidence rate of anorexia nervosa for the presumed peak age of risk (10–24y) was 15.7 per 100 000 person-years; its lifetime prevalence was 0.24%. All probands had recovered from eating disorders, but suffered from substantial psychiatric comorbidity, which also manifested in their co-twins. Additionally, male co-twins displayed significant dissatisfaction with body musculature, a male-specific feature of body dysmorphic disorder.

**Conclusions:**

Anorexia nervosa in males in the community is more common, transient and accompanied by more substantial comorbidity than previously thought.

## Introduction

Believed to be rare, little is known about anorexia nervosa in males. Few studies have reported on its incidence, prevalence, comorbidity, or familial aggregation [1]–[3]. The risk for the illness in women peaks at age 10–24: similar peak age of risk appears likely in men [4].

The contemporary sociocultural environment of developed countries is generally more conducive to anorexia nervosa in women than men. It has been hypothesized that to develop anorexia nervosa in this context, men require a greater loading of genes or adverse environmental factors than women [5]. In women, anorexia nervosa shares a familial diathesis with affective and anxiety disorders [6], [7]. Body dysmorphic disorder and eating disorders show intra-individual clustering within both women and men [8], but little is known about the familial co-aggregation of these conditions.

The present study used a large population based cohort of twins to examine the incidence and prevalence of anorexia nervosa in males; comorbid conditions in anorexia nervosa probands; and comorbid conditions in the co-twins of the probands.

## Methods

### Ethics Statement

Data collection and analysis was approved by the ethics committee of Helsinki University and of the Institutional Review Board at Columbia University.

### Sample

**Figure 1:**
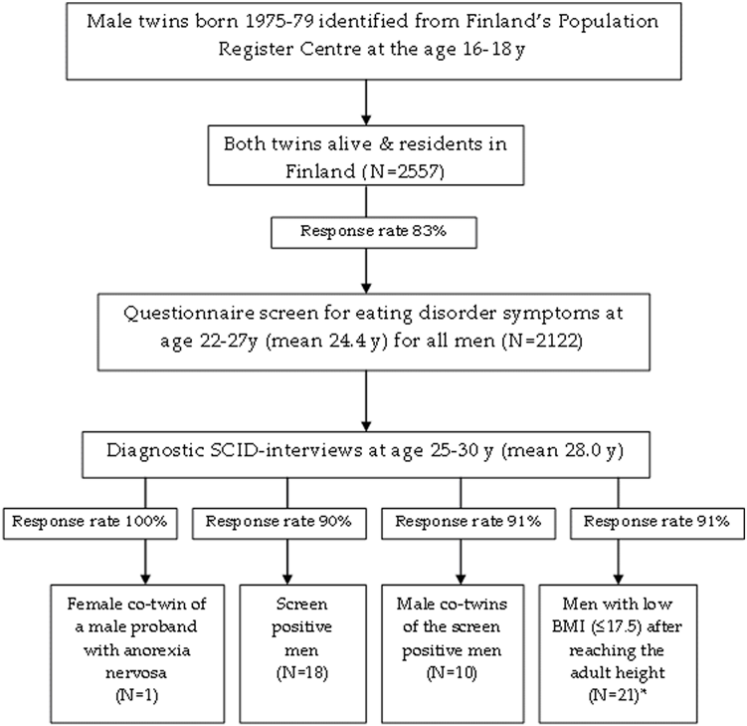
Flow chart of the data collection of eating disorder study in males of FinnTwin16 cohorts. * Excluded N = 10 men whose low BMI resulted from missing limbs or tetraplegia; or other severe physical illness (multiple sclerosis, cerebral palsy).

Virtually all twins born in 1975–79 were identified from the Finnish population register [9] and assessed at ages 16, 17, 18 and 22–28y. The design of this study was modelled after a comparable study conducted among women described in detail elsewhere [10]. Flow chart of the data collection of eating disorder study in males of FinnTwin16 cohorts is shown in the Figure 1. Briefly, at age 22–27y (mean 24.4y, SD 0.83), we screened by questionnaire all men (N = 2557; response rate of 83% yielded N = 2122) for eating disorder symptoms. We also assessed their current self-reported height and minimum, maximum and current self-reported weight after reaching the adult height, from which we calculated their body mass indexes (BMI). In a sub-sample of 133 men, not selected for eating disorders or eating behavior, the correlation between self-reported and later measured BMI was 0.91 [11].

Men who reported or suspected ever having had an eating disorder (N = 20) and their male co-twins (N = 11) were invited to participate in diagnostic telephone interviews. After confirming an eating disorder in a screen-positive man who had a female co-twin, she was also interviewed. Further, 33 men who reported a minimum BMI ≤17.5 after reaching their adult height were invited to the interview, excluding men (N = 10) whose low BMI resulted from serious physical disability (Figure 1).

All participants gave an oral informed consent after being provided with information about the study. In addition, all but one of the probands and all their co-twins signed a written informed consent for this study. One proband gave an oral informed consent, but could not be reached for the signature.

### Diagnostic interviews

Two MDs from the Eating Disorder Unit of Helsinki University Central Hospital conducted the short version of the Structured Clinical Interview (SCID) for DSM-IV [12] to obtain current and lifetime diagnoses of anorexia nervosa, bulimia nervosa, binge-eating disorder, major depression, and obsessive-compulsive disorder and to define the ages of onset and resolution of symptoms, and the temporal sequence and time course of these diagnoses. The interviewers also assessed symptoms of body dysmorphic disorder and male-specific body image concerns, including muscle dissatisfaction and muscle dysmorphia, and muscle building supplement and anabolic hormone use. The interviews were conducted for the screen positive men in a way that rendered questions exactly similar for all respondents.

The interview participation rate for males was 90.7% (Figure 1). None of the selected individuals refused to be interviewed, but five of them could not be reached. One of the probands with anorexia nervosa had a female co-twin and she participated.

### Definition of disorder

We used DSM-IV [13] criteria for anorexia nervosa, excluding amenorrhea. The weight criterion (A) was based on ≤85% of expected body weight and BMI below the tenth percentile for an age-matched Finnish twin population [14], or significant (≥20%) weight loss leading to underweight.

### Prevalent and incident cases

We defined lifetime prevalent cases to include the cases identified in this study, regardless of age at onset, age at interview, or recovery. Lifetime prevalence was calculated by dividing the number of lifetime prevalent cases by the number (N = 2122) of responders at age 22–27 y with binomial confidence intervals. We computed incidence for the age of risk 10–24.9 years, based on age of onset in cases and the total number of person-years at risk, using Poisson-based confidence intervals.

## Results

### Prevalence and incidence

We found five participants with DSM-IV lifetime anorexia nervosa (Table 1). All probands were screen positive in the eating disorder screen. Lifetime prevalence rate was 0.24% (95% CI 0.03–0.44%). The incidence rate for 10–24.9 y was 15.7 (95% CI 6.6–37.8) per 100 000 person-years.

**Table 1:** Characteristics of the male anorexia nervosa probands and their co-twins in the population-based FinnTwin16 study.

Twin pair	Zygosity	Proband, age of AN onset	Proband, Min BMI	Proband, lifetime comorbid diagnoses and sub-diagnostic symptoms	Co-twin, lifetime diagnosis and sub-diagnostic symptoms
I	Monozygotic Male-Male	23	16.4	Major Depression, Obsessive Compulsive Disorder, Muscle Dissatisfaction, Muscle Building Supplement Use	Bipolar Affective Disorder, Sub-diagnostic Obsessive Compulsive Disorder, Muscle Dissatisfaction, Muscle Building Supplement Use
II	Dizygotic Male-Male	17	15.1	Major Depression	Muscle Dissatisfaction, Muscle Building Supplement Use
III	Dizygotic Male-Male	15	14.7	Major Depression	Major Depression
IV	Dizygotic Male-Male	17	18.4	Bulimia Nervosa (P^1^)	Major Depression, Obsessive Compulsive Disorder, Muscle Dissatisfaction, Muscle Building Supplement Use
V	Dizygotic Male-Female	14	18.6	Major Depression, Bulimia Nervosa (NP^2^)	Major Depression

*Note*: ^1^Purging,^ 2^Non-purging

### Course and features of illness

Median age at the onset of anorexia nervosa was 17.0 y. The mean minimum BMI of men with the illness was 16.6 kg/m^2^ (range 14.7–18.6 kg/m^2^). Before the illness onset, all five probands had been overweight (BMI range 23.9–30.4 kg/m^2^) according to the height-specific Finnish growth charts (Helsinki University Hospital for Children and Adolescents). After on average 1.6 years, all probands had recovered (defined as restoration of weight, absence of intense fear of weight gain and of bingeing and purging for at least 1 year prior to assessment). Two had received treatment for anorexia nervosa.

### Psychiatric comorbidity

All but one of the probands had suffered from DSM-IV major depression, three of them from more than one episode (Table 1). In all four, anorexia nervosa preceded the onset of major depression. One proband had a DSM-IV obsessive compulsive disorder. Cross-over to bulimia nervosa occurred in two probands: proband V suffered from non-purging bulimia for two years, and proband IV suffered from purging-type bulimia for three years. After anorexia nervosa, one proband suffered from symptoms of body dysmorphic disorder (high muscle dissatisfaction, excessive gym training and muscle building supplement use).

### Co-twins

All participants were discordant for anorexia nervosa within the twin pair. However, the co-twins displayed significant psychopathology (Table 1): three experienced symptoms of body dysmorphic disorder (significant muscle dissatisfaction, frequent gym-training, and established use of muscle building supplements), but none fulfilled the full diagnostic criteria for it. One co-twin had current DSM-IV major depression, while two had a history of major depression and one co-twin suffered from bipolar disorder. One of the co-twins had DSM-IV obsessive-compulsive disorder, and another displayed obsessive compulsive symptoms that remained below the diagnostic threshold. The current mean BMI of the co-twins during the interview was 25.0 (range 19.4–34.5 kg/m^2^); their mean minimum BMI was 20.3 kg/m^2^ (range 18.5–22.9 kg/m^2^) and mean maximum BMI was 26.4 kg/m^2^ (range 19.4–34.5 kg/m^2^). None of them had been overweight in adolescence.

## Discussion

Our study has four important findings. First, although anorexia nervosa in adolescent and young men from the community was transient, it was accompanied by significant psychiatric comorbidity, which also manifested in their co-twins. The pattern of common familial diathesis of anorexia nervosa and of affective and anxiety disorders [6], [7] as well as of obsessive-compulsive personality disorder [15] has earlier been demonstrated only among females in the clinical samples. Interestingly, the co-twins of the male probands differed remarkably from those of female probands in the same twin cohort, whose co-twins were mainly healthy [10], which supports the hypothesis presented by Strober et al. [5] suggesting that in order to develop an eating disorder, men require a stronger genetic predisposition and/or more adverse environmental factors than women. Second, compared to young males from the general population [16], symptoms of body dysmorphic disorder were frequent in the co-twins, suggesting a common familial diathesis for anorexia nervosa and body dysmorphic disorder. Third, anorexia nervosa in young men was more common than suggested by previous reports. The most age comparable study was in the UK and based on the General Practice Database [4]; it reported significantly lower incidence rates than our study (2.3 for 10–19 y and 0.5 per 100 000 person years for 20–39 y). Studies in Sweden [1], Canada [2] and the US [3] also reported lower life-time prevalence of DSM-IV anorexia nervosa in young men, but are not readily comparable to ours because of varying age of the participants (up to 67 y) at the time of assessment and thus potentially long retrospective recall. In contrast, the incidence of anorexia nervosa among women aged 10–24y in our twin cohorts was 140 per 100 000 person years, which is approximately nine-fold compared to men (Dr. Keski-Rahkonen, personal communication). Finally, as previous studies suggest, we found that also in our study, every proband with anorexia nervosa had been premorbidly overweight.

According to our study, approximately 1 out of 400 boys suffer from anorexia nervosa by young adulthood. However, the illness in males is rarely encountered in clinical settings, perhaps due to short illness duration, treatment delay, and fear of social consequences of diagnosis. The higher than expected prevalence must be interpreted with caution, because of the limited number of cases of anorexia nervosa, and of wide confidence limits. However, it is liable that the sensitivity of our screen was sub-optimal; thus the prevalence of 0.24% is more likely to be an underestimate than an overestimate of the true prevalence of anorexia nervosa in the male population. Most of the screen positive men in our sample had some eating problems, but not a diagnosable eating disorder. A couple of men who reported that they had an eating disorder actually suffered from other severe mental illnesses, such as schizophrenia. Surprisingly, we did not detect any cases of bulimia nervosa without previous anorexia nervosa.

To our knowledge, this is the first study to report psychiatric morbidity of the co-twins of the male probands with anorexia nervosa. Our findings of the intra-individual and intra-familial clustering of anorexia nervosa, affective disorders and symptoms of obsessive compulsive disorder support the earlier findings of shared biological vulnerability for these disorders. However, the contrast in the mental health of co-twins of the female [10] *versus* male probands in our sample also suggests that in the general population, men who develop anorexia nervosa may exhibit stronger vulnerability for the illness compared to women. In addition, at least in a speculative level, these findings suggest that in men, the underlying vulnerability might manifest either as an eating disorder or as (symptoms of) body dysmorphic disorder, e.g. muscle dissatisfaction and muscle building substance use. However, because of a small number of cases and the co-twins, we suggest caution when interpreting these findings.

The strengths of our study include assessment at the end of the peak age of risk, good population coverage, excellent participation, interviews by eating disorder experts and co-twin controls. Limitations include small number of detected cases, self-report bias and recall bias; both at screening and at the diagnostic interview, self-reported figures of weight and height might have been biased due to the potential delicate nature of these measures among individuals with eating problems. In addition, less than perfect sensitivity of our screen may have resulted, had some participants concealed their eating disorders or other related problems due to the fear of stigmatization. Although difficult to overcome, we highly recommend that future studies specifically address this issue in their study designs in order to reach as many male probands as possible.

### Conclusions

In our study, anorexia nervosa in boys and young men from the general population was more common, transient and accompanied by more substantial co-morbidity than previously thought. Co-twins of the probands displayed significant psychopathology such as male specific symptoms of body dysmorphic disorder, but none of them had had an eating disorder. Future studies should examine psychiatric morbidity of the first-degree relatives of males with anorexia nervosa in large, preferably population-based samples to replicate our findings in the co-twins.

All authors report no competing interests.
